# Quantum Error Mitigation in Optimized Circuits for Particle-Density Correlations in Real-Time Dynamics of the Schwinger Model

**DOI:** 10.3390/e27040427

**Published:** 2025-04-14

**Authors:** Domenico Pomarico, Mahul Pandey, Riccardo Cioli, Federico Dell’Anna, Saverio Pascazio, Francesco V. Pepe, Paolo Facchi, Elisa Ercolessi

**Affiliations:** 1 Dipartimento di Fisica, Università di Bari, I-70126 Bari, Italy; domenico.pomarico@ba.infn.it (D.P.); saverio.pascazio@ba.infn.it (S.P.); paolo.facchi@ba.infn.it (P.F.); 2 Istituto Nazionale di Fisica Nucleare, Sezione di Bari, I-70126 Bari, Italy; 3 Istituto Nazionale di Fisica Nucleare, Sezione di Bologna, I-40127 Bologna, Italy; federico.dellanna2@unibo.it (F.D.); elisa.ercolessi@unibo.it (E.E.); 4 Dipartimento di Fisica e Astronomia, Università di Bologna, I-40127 Bologna, Italy

**Keywords:** noisy intermediate-scale quantum devices, circuit optimization, error mitigation, quantum electrodynamics

## Abstract

Quantum computing gives direct access to the study of the real-time dynamics of quantum many-body systems. In principle, it is possible to directly calculate non-equal-time correlation functions, from which one can detect interesting phenomena, such as the presence of quantum scars or dynamical quantum phase transitions. In practice, these calculations are strongly affected by noise, due to the complexity of the required quantum circuits. As a testbed for the evaluation of the real-time evolution of observables and correlations, the dynamics of the $Z_{n}$ Schwinger model in a one-dimensional lattice is considered. To control the computational cost, we adopt a quantum–classical strategy that reduces the dimensionality of the system by restricting the dynamics to the Dirac vacuum sector and optimizes the embedding into a qubit model by minimizing the number of three-qubit gates. The time evolution of particle-density operators in a non-equilibrium quench protocol is both simulated in a bare noisy condition and implemented on a physical IBM quantum device. In either case, the convergence towards a maximally mixed state is targeted by means of different error mitigation techniques. The evaluation of the particle-density correlation shows a well-performing post-processing error mitigation for properly chosen coupling regimes.

## 1. Introduction

Investigations of lattice gauge theories constitute an interface among fundamental physics, the characterization of quantum many-body systems, and quantum computing implementations by means of properly engineered coding strategies [1,2]. The availability of noisy intermediate-scale quantum (NISQ) devices in cloud access platforms opens interesting possibilities from both academic and industrial perspectives. Nonetheless, the operating quantum systems are critically affected by noise, thus preventing current NISQ devices from actually outperforming classical computing capabilities [3]. This statement holds true in a diversified way in the cases of different hardware setups engineered for quantum computing purposes, each characterized by different advantages in terms of gate fidelity and experimental realization.

As a benchmark, in this article, we concentrate on the lattice version of quantum electrodynamics (QED) in one dimension, the so-called Schwinger model [4]. A rich variety of phenomena emerge, driven by the intrinsic nature of lattice QED as a kinetically constrained model, due to the existence of conditions that limit the space of physical states [5,6,7]. This characterization leads to the observation of quantum scarring, preserved through linear gauge protection [8,9,10,11] and detected by means of out-of-time-ordered correlators [12]. Other relevant observables are represented by non-equal-time correlation functions, used to detect non-analyticities that point out dynamical quantum phase transitions (DQPTs) in quantum quenches [13,14,15,16,17,18,19,20]. In this context, particle density is specifically relevant, as it can represent the most intuitive quantity to describe in the continuum limit, the decay of an initial Dirac vacuum state after a quench [21,22].

A first attempt to simulate the dynamics of one-dimensional QED was implemented with ion traps [21,23], targeting the observation of pair production starting from the Dirac vacuum. Further developments adopted quantum–classical algorithms in superconducting circuits, based on embedding the dynamics in a specific vacuum sector [24]. Larger system sizes were investigated through analog simulators in optical lattices that host ultracold atoms [25,26]. In setups accessible via cloud platforms, noise that affects computation [27,28,29,30,31,32,33,34] can limit the observation of targeted phenomena [35,36], and error mitigation techniques must be implemented to recover physically meaningful results [37,38,39,40,41,42,43,44].

Our investigation directly targets the possibility to recover the expectation values and correlation functions of physical quantities in spite of a fast convergence towards a maximally mixed state, when working in digital mode. To face the increased experimental complexity measured in terms of circuit depth, we propose an algorithm that makes use of a classical–quantum procedure to optimize the embedding of our model into a qubit system. Specific implementations of Trotter evolution are further used to evaluate particle-density correlation functions for the Schwinger model, based on specifically engineered circuits exploiting an ancilla qubit as proposed for general retarded Green functions [45,46,47]. This lattice QED use case scenario constitutes a properly suited test for error mitigation strategies [48,49] and the related post-processing capabilities in managing noise to evaluate physically meaningful quantities. A non-equilibrium quench protocol is described to study time evolutions of the initial Dirac vacuum characterized by pair production, signaling the emergence of a new ground state, eventually leading to a new phase for a proper choice of parameters [50]. The study of lattice size scaling is beyond the scope of this article, while we focus on possible methodological and circuit tools that are useful in NISQ devices in the characterization of non-equilibrium dynamics, including critical conditions which are hardly captured experimentally [35].

The content is as follows. In Section 2, we give a brief introduction to the Schwinger model on a one-dimensional lattice and with a discretized gauge symmetry group, which is mapped into a quantum spin system via a Jordan–Wigner transformation. In Section 3, we adopt a quantum–classical embedding of the considered dynamics by first restricting the dynamics in the Dirac vacuum sector by means of translation and charge conjugation symmetries and then choosing the optimal permutation of states that minimizes the number of three-qubit interactions in the Hamiltonian. Then, in Section 4, we describe the observables we are interested in, namely particle-density operators and their correlation functions, and derive the digital circuits that implement their real-time evolution. Section 5 presents our results, run for a lattice composed by N=4 sites, comparing exact evolution, bare noisy simulations and simulations with error mitigation techniques, such as twirled readout error extinction (T-REx) [48] and zero noise extrapolation (ZNE) [49]. For the evolution of the particle-density operators, we also perform runs on a physical IBM device. Finally, we draw our conclusions in Section 6 and collect some details of our calculations in Appendix A, Appendix B, Appendix C, Appendix D, Appendix E and Appendix F.

## 2. Lattice QED in One Spatial Dimension

Lattice QED in (1+1) dimensions, representing the spatial discretization of the Schwinger model, is a prototypical case of a gauge theory formulated in reduced spatial dimensionality. In this model, gauge degrees of freedom consist of a single (longitudinal) component of the electric field, interacting with spinless fermions of mass *m* and charge *g*: canonically anticommuting operators $\psi_{x},\;\psi_{x}^{†}$ that represent the matter field live on sites *x*, while each edge with length *a* connecting sites *x* and x+1 hosts the electric field operator $E_{x,x+1}$ and the gauge connection $U_{x,x+1}=e^{iaA_{x,x+1}}$, where $A_{x,x+1}$ is the vector potential, canonically commuting with the electric field. Since we consider a finite lattice with *N* sites, labeled by x∈{0,…,N−1}, we require periodic boundary conditions and identify the fields corresponding to the index x=N to those at the x=0 boundary. The system evolution is determined by the Hamiltonian

(1) $$H=-\frac{iJ}{2}\sum_{x=0}^{N-1}\left(\psi_{x}^{†}U_{x,x+1}\psi_{x+1}-H.c.\right)+m\sum_{x=0}^{N-1}{\left(-1\right)}^{x}\psi_{x}^{†}\psi_{x}+\frac{g^{2}}{2J}\sum_{x=0}^{N-1}E_{x,x+1}^{2}.$$

The first term represents the nearest-neighbor hopping of fermions accompanied by the corresponding transformation of the electric field on the involved lattice edge [21,23,35] with $J=a^{-1}$, the second term is the staggered (Kogut–Susskind) mass term, which is able to solve the doubling problem associated to lattice discretization [51], while the last term gives the standard electric contribution to energy. The Dirac vacuum state, which is realized by creating one fermion in each of the negative-mass (odd-*x*) sites, coincides with the ground state in the limit of infinite mass [16,17]. We adopt a quench protocol based on an initial Dirac vacuum with a time evolution generated by a Hamiltonian with finite mass such that the initial state is no longer a ground state [13,14,15,16,17,18,19,20]. We notice that Hamiltonian (1) is invariant under charge conjugation and translation by two lattice sites, which, for a lattice with even number *N* of sites [50], read as

(2) $$\begin{array}{cc} C_{+}= & \;\left\{\begin{array}{cc} \psi_{x}\to {\left(-1\right)}^{x+1}\psi_{x+1}^{†},\;\; & \psi_{x}^{†}\to {\left(-1\right)}^{x+1}\psi_{x+1},\\ E_{x,x+1}\to -E_{x+1,x+2},\;\; & U_{x,x+1}\to U_{x+1,x+2}^{†}, \end{array}\right. \end{array}$$

(3) $$\begin{array}{cc} T_{2}= & \;\left\{\begin{array}{cc} \psi_{x}\to \psi_{x+2},\;\; & \psi_{x}^{†}\to \psi_{x+2}^{†},\\ E_{x,x+1}\to E_{x+2,x+3},\;\; & U_{x,x+1}\to U_{x+2,x+3}, \end{array}\right. \end{array}$$

(4) $$\begin{array}{cc} \lambda (C_{+})= & \;\left\{+1,-1,+i,-i\right\},\;\lambda \left(T_{2}\right)=\left\{+1,-1\right\}, \end{array}$$

where λ denotes the spectrum, $C_{+}$ stands for a transformation translating matter and electric fields of a lattice spacing (from site *x* to site x+1), transforming particles into antiparticles and vice versa, while changing the sign to the electric field. $C_{-}$ acts in the opposite direction. We also set $C_{-}=C_{+}^{†}$ with translation by two lattice sites $T_{2}=C_{+}^{2}$.

A further discretization, involving the local gauge degrees of freedom, consists of replacing the original U(1) gauge group with the finite cyclic group $Z_{n}$. Thus, the Hilbert space associated to a given link becomes *n*-dimensional [52]. In this case, we have the following [50,52]:

1. A convenient basis of the edge space is represented by the electric field eigenstates $\left\{|e_{k}\rangle \right\}$, satisfying $E_{x,x+1}|e_{k}\rangle =e_{k}|e_{k}\rangle$, with $e_{k}=\sqrt{\frac{2\pi }{n}}\left(k-\frac{n-1}{2}\right)$ and k=0,…,n−1;
2. The gauge connection $U_{x,x+1}$ acts on this basis as a cyclic permutation: $U_{x,x+1}|e_{k}\rangle =|e_{k+1}\rangle$ and $U_{x,x+1}|e_{n-1}\rangle =|e_{0}\rangle$;
3. The lattice counterpart of the Gauss law $G_{x}|\phi \rangle =0$ which fixes the admissible states |ϕ〉 is given by
  (5) $$G_{x}=\sqrt{\frac{n}{2\pi }}\left(E_{x,x+1}-E_{x-1,x}\right)-\psi_{x}^{†}\psi_{x}-\frac{{\left(-1\right)}^{x}-1}{2},$$
  which must be valid at all sites *x* and at any time.

To take on quantum computation on the model Hamiltonian (1), it is convenient to perform the Jordan–Wigner transformation

(6) $$\psi_{x}=\sigma_{x}^{+}\prod_{\ell =0}^{x-1}\left(-iZ_{\ell }\right),\;\mathrm{with}\;\sigma_{x}^{\pm }=\frac{X_{x}\pm iY_{x}}{2},$$

where $X_{x}$, $Y_{x}$ and $Z_{x}$ are Pauli matrices acting on the state of site *x* in the fermion occupation number basis. The transformation maps the matter field into a collection of *N* spins (qubits), with the advantage of working with operators that commute at different sites. We also rescale the transformed Hamiltonian [19,20,21,23,24,35] by setting $\xi =J^{2}/g^{2}$, $\mu =mJ/g^{2}$ to finally obtain the Hamiltonian rewritten as

(7) $$H\left(\xi ,\mu \right)=\xi \sum_{x=0}^{N-1}\left(\sigma_{x}^{-}U_{x,x+1}\sigma_{x+1}^{+}+H.c.\right)-\mu \sum_{x=0}^{N-1}{\left(-1\right)}^{x}Z_{x}+\sum_{x=0}^{N-1}E_{x,x+1}^{2},$$

where for fermionic hopping terms wrapping around the boundary $-i\psi_{N-1}^{†}U_{N-1,0}\psi_{0}=i^{N}\left(\prod_{\ell =0}^{N-1}Z_{\ell }\right)\sigma_{N-1}^{-}U_{N-1,0}\sigma_{0}^{+}$, with the same sign of $i^{N}$ and $\prod_{\ell =0}^{N-1}Z_{\ell }$ for any *N* at half filling.

In the following, we consider a lattice composed by N=4 sites with two particles. We also discretize the unitary group with $Z_{3}$. Appendix A contains a description for the gauge-invariant subspace and the orbits of the charge conjugation and translation operators for this case, which identify the basis required to explicitly highlight the Hilbert space sectors.

## 3. Classical–Quantum Embedding

In this section, we present the algorithm we use to simulate the dynamics of the model described by the Hamiltonian (7). To have an efficient protocol, it is necessary to reduce the number of quantum resources needed for the simulation. We do so by adopting a two-step scheme: first we restrict the computation to the subspace containing the Dirac vacuum, and then we consider a qubit embedding in which the Hamiltonian contains the smallest number of three-body terms.

To reach the first goal, we split the Hilbert space by considering the translation by two lattice sites and charge conjugation symmetry sectors, which are labeled by their eigenvalues $T_{2}=+1$ and C=±1, $T_{2}=-1$ and C=±i listed in Equation (4). It is thus possible to partition the physical Hilbert space into four sectors such that the Hamiltonian (7) is expressed as $UHU^{†}={⨁}_{T_{2},C}H^{\left(T_{2},C\right)}$. We exploit the block structure described in Appendix A to focus on the (+,+) sector, containing the initial Dirac vacuum. The restriction of the Hamiltonian to this seven-dimensional subspace reads

(8) $$H^{\left(+,+\right)}=\left(\begin{array}{ccccccc} -2\mu & \xi & \; & & \; & & \\ \xi & \frac{\pi }{3} & \frac{\xi }{\sqrt{2}} & \; & & \; & \\ \; & \frac{\xi }{\sqrt{2}} & 2\mu +\frac{2\pi }{3} & \frac{\xi }{\sqrt{2}} & \; & & \\ \; & \; & \frac{\xi }{\sqrt{2}} & \pi & \frac{\xi }{\sqrt{2}} & \; & \\ \; & \; & & \frac{\xi }{\sqrt{2}} & -2\mu +\frac{4\pi }{3} & \frac{\xi }{\sqrt{2}} & \\ \; & \; & & \; & \frac{\xi }{\sqrt{2}} & \frac{4\pi }{3} & \xi \\ \; & \; & & \; & & \xi & 2\mu +\frac{4\pi }{3} \end{array}\right),$$

according to the ordered basis given in Appendix A.

As for the embedding, we notice that seven states can be described by means of three qubits, the elementary approach relying on adding a last column and row filled with zeros to the Hamiltonian matrix to keep the eighth unphysical state idle. The direct approach consists in identifying the first seven states of a three qubit system with the seven states (A1)–(A7) listed in Appendix A: doing so, the Hamiltonian (8) can be written as

(9) $${\tilde{H}}^{\left(+,+\right)}=\frac{1}{8}\sum_{i,j,k=0}^{3}\mathrm{Tr}\left\{\sigma_{i}⊗\sigma_{j}⊗\sigma_{k}{\tilde{H}}^{\left(+,+\right)}\right\}\sigma_{i}⊗\sigma_{j}⊗\sigma_{k}=\sum_{i,j,k=0}^{3}c_{\left(i,j,k\right)}\sigma_{i}⊗\sigma_{j}⊗\sigma_{k},$$

where $\sigma_{0}$ is the 2×2 identity matrix, and $\sigma_{j}$ with j∈{1,2,3} are the Pauli matrices. Here and in the following, we use the tilde notation to emphasize the specific embedding into the three-qubit system. A straightforward but lengthy calculation shows that such a decomposition accounts for 19 non-zero terms, of which 7 correspond to a product of 3 Pauli matrices.

In the implementation of the Trotter evolution for $U\left(t\right)=\exp\left(-i{\tilde{H}}^{\left(+,+\right)}t\right)$, the latter terms require a higher number of CNOT gates. Thus, in order to reduce the computational time, we aim at finding the embedding in which the Hamiltonian contains the smallest number of three-body terms, i.e., we want to minimize the number of coefficients $c_{\left(i,j,k\right)}$ with i,j,k∈{1,2,3} by considering all possible permutations $\pi \in S_{8}$ of the eight basis states. For the chosen problem, we find that the optimal permutation is $\pi_{o}=\left(7,6,1,2,4,5,8,3\right)$, leading to a Hamiltonian ${\tilde{H}}_{\pi_{o}\left(\ell \right),\pi_{o}\left(m\right)}^{\left(+,+\right)}$ which contains 15 non-zero terms, of which only 3 are triple products of Pauli matrices gates. In Appendix B, we list all coefficients for both the simple and the optimal embedding in the case we consider here, while in Appendix C, we propose an algorithm to find such an optimal permutation in the general case. Finally, to reduce the usage of SWAP gates, circuit optimization can also rely on a properly chosen topology of superconducting qubit connections [35].

## 4. Digital Circuit for Unitary Evolution and Green Functions

**Figure 1:**
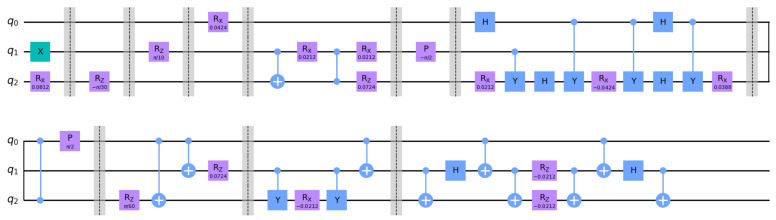
Trotter step circuit for the permutation $\pi_{o}=\left(7,6,1,2,4,5,8,3\right)$ and Δt=0.1. ξ=0.6, μ=0.1, with the initial spin flip required to map ${|\mathrm{vac}\rangle }_{i}\to {|\mathrm{vac}\rangle }_{\pi_{o}\left(i\right)}$.

Once we have obtained the optimal decomposition for the Hamiltonian, the time-evolution operator U(t) is implemented via a Trotter decomposition as described in Appendix D. Each Trotter step is therefore represented by the circuit shown in Figure 1.

Let us look now at observables of interest. The ones we want to consider are the time evolution of particle density and its associated correlation functions. The formal expression of the former is [21]

(10) $$\nu =\frac{1}{N}\sum_{x}{\left(-1\right)}^{x}\psi_{x}^{†}\psi_{x},$$

admitting the sector decomposition $U\nu U^{†}={⨁}_{T_{2},C}\nu^{\left(T_{2},C\right)}$, once we restrict it to the physical subspace. In particular, in the (+,+) sector, we have

(11) $${\tilde{\nu }}^{\left(+,+\right)}=\sum_{i,j,k=0}^{3}d_{\left(i,j,k\right)}\sigma_{i}⊗\sigma_{j}⊗\sigma_{k},$$

with a number of non-vanishing coefficients that depends on the specific permutation we choose for the embedding. In Appendix B, we list the non-vanishing coefficients for the optimal permutation described above and give the explicit expression (A20) that we use in the following calculations.

The real-time correlation function is defined as

(12) $$G\left(t,s\right)=G^{<}\left(t,s\right)-G^{>}\left(t,s\right)=-i\Theta \left(t-s\right)\langle \mathrm{vac}|\{{\tilde{\nu }}^{\left(+,+\right)}\left(t\right),{\tilde{\nu }}^{\left(+,+\right)}\left(s\right)\}|\mathrm{vac}\rangle ,$$

where, for any two operators, {A,B}=AB+BA and Θ(t) is the Heaviside step function. We can choose two arbitrary times s,t since the correlation function vanishes for s=0 because of ${\tilde{\nu }}^{\left(+,+\right)}\left(0\right)|\mathrm{vac}\rangle =0$. G(t,s) is composed of two terms, named the lesser $G^{<}\left(t,s\right)$ and the greater term $G^{>}\left(t,s\right)$. The former quantifies for an initial time *s* how long the particle-density property persists in the future, while the latter measures this memory effect backward in time.

Here, we focus on the lesser term $G^{<}\left(t,s\right)$ in order to achieve a trade-off between hardware complexity and physical interpretability. To be precise, the main source of error is the sum of individual decoherence effects imposed by the large number of controlled double-qubit gates as presented in Appendix D. Adding the greater term $G^{>}\left(t,s\right)$ would greatly affect the mitigating action by introducing too many noise sources, while not adding considerably to the physical insights gained from the simulation.

The lesser term is defined as

(13) $$G^{<}\left(t,s\right)=-i\Theta \left(t-s\right)\langle \mathrm{vac}|{\tilde{\nu }}^{\left(+,+\right)}\left(t\right){\tilde{\nu }}^{\left(+,+\right)}\left(s\right)|\mathrm{vac}\rangle ,$$

which, using the definition of $P_{\alpha }$ in Equation (A20) and the explicit value of the coefficient $d_{\left(0,0,0\right)}=7/16$, can be rewritten as

(14) $$\begin{array}{cc} G^{<}\left(t,s\right)=-i\Theta \left(t-s\right) & \left[\frac{7}{16}\left(\langle \mathrm{vac}|{\tilde{\nu }}^{\left(+,+\right)}\left(t\right)|\mathrm{vac}\rangle +\langle \mathrm{vac}|{\tilde{\nu }}^{\left(+,+\right)}\left(s\right)|\mathrm{vac}\rangle \right)-\frac{49}{256}\right.\\ \; & \;\left.+\sum_{\alpha ,\beta =1}^{7}\langle \mathrm{vac}|U^{†}\left(t\right)P_{\alpha }U\left(t-s\right)P_{\beta }U\left(s\right)|\mathrm{vac}\rangle \right]. \end{array}$$

The first two terms of this expression are the expectation values of the density operator evaluated on the time-evolved vacuum state and can be evaluated using the Trotter decomposition of the evolution operator and the circuit of Figure 1. The last term can be instead calculated via the circuit [46](15)
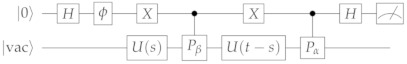

which uses a measurement over an ancilla qubit *A* (initialized in the state |0〉). Indeed, denoting with *R* the register encoding the system (initialized in the vacuum state |vac〉) and with $\varrho_{\mathrm{out}}$ the output state of the total circuit, it is not difficult to prove that the measurement on the ancilla yields

(16) TrAR(Z⊗1)ϱout=Ree−iϕ〈vac|U†(t)PαU(t−s)PβU(s)|vac〉.

In Appendix E, we discuss how this circuit can be realized by means of a Mach–Zender interferometer [53,54].

## 5. Implementation on IBM Quantum Platform

The algorithm described in the previous section is implemented on the IBM Quantum platform [55], more specifically both on the device ibmq_quito and by means of noise models available in the Python package qiskit (https://pypi.org/project/qiskit/, accessed on 10 April 2025), with error rates updated from the aforementioned device. To limit noise sources in state preparation, gates and measurements, error mitigation techniques are applied in both cases using tools of the package qiskit-ibm-runtime as discussed in Appendix F. In the following, we present the results for the problem of N=4 sites, a chosen number of 1000 shots, whose collected statistics impose the shown error bars, and the available LinearExtrapolator for ZNE.

**Figure 2:**
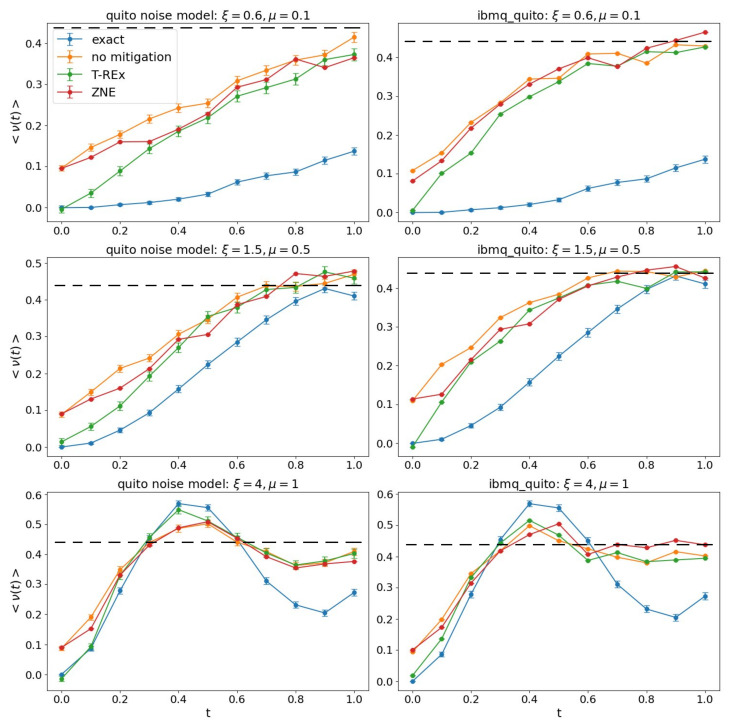
Results for the chosen optimal permutation π=(7,6,1,2,4,5,8,3) and Trotter step Δt=0.1. Panels in the left column refer to ibmq_quito noise model, while those in the right one refer to the device ibmq_quito measurements. The three rows correspond to the three different coupling regimes (ξ,μ)={(0.6,0.1),(1.5,0.5),(4,1)} from top to bottom. The dashed line stands for the maximally mixed condition ν=7/16, while the color of solid dotted lines is explained in the legend. From top to bottom: strong to weak coupling.

The real-time evolution of the particle density (11) is shown in Figure 2, with the first column showing the noise-simulated data and the second column giving the output of the actual computation on the device. Since the initial state corresponds to the Dirac vacuum, we can interpret the considered simulation as a quenched dynamics starting from the ground state for μ→∞ in (7), followed by an evolution generated for finite μ and ξ. We call the strong (weak) coupling regime a condition in the free parameters space (ξ,μ) that causes a slowly (rapidly) deviating evolution from the initial Dirac vacuum. The three rows of Figure 2 correspond to three different coupling regimes that go from strong to weak coupling, that is, (ξ,μ)={(0.6,0.1),(1.5,0.5),(4,1)}. In each graph, we compare four curves: the exact noiseless simulation (in blue), the bare results of noisy simulation/real results (in orange), and the results processed via two error mitigation techniques, T-REx (in green) and ZNE (in red).

The ZNE mitigation is characterized by an almost identical curve with the unmitigated case, while the T-REx mitigation works better during the first time steps, where noise is mainly dominated by readout errors. We observe that the behavior of noisy and mitigated curves are more affected in the strong coupling case. However, in all cases, for longer times, the expectation value approximates the maximally mixed condition $\langle {\tilde{\nu }}^{\left(+,+\right)}\left(t\right)\rangle =\frac{7}{16}$, showing that noise destroys any coherence. Also, for the real device ibmq_quito, the strong and intermediate couplings converge faster towards the maximally mixed condition than the corresponding noise model, probably because of the missing correlated noise in the assumed standalone gate hypothesis [35], which neglects, for example, the noise propagation in subsequent applications of double qubit gates sharing a circuit line. The resilient behavior of the weak coupling regime may be due to the presence of the ξ parameter in all double and triple qubits gates presented in Appendix B.

**Figure 3:**
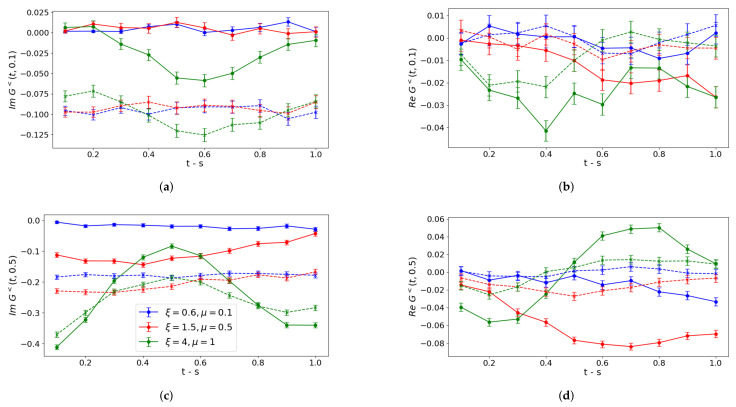
Bare noise simulation. Lesser contribution to correlation functions of particle densities ${\tilde{\nu }}^{\left(+,+\right)}\left(t\right)$ and ${\tilde{\nu }}^{\left(+,+\right)}\left(s\right)$ for a non-vanishing time *s*: s=0.1 in (**a**,**b**), s=0.5 in (**c**,**d**). Circuits are executed in qasm_simulator. Solid lines correspond to noiseless Trotter evolution with error bars caused by the finite number of shots, while dashed lines include noise models. Different colors refer to different coupling regimes (see panel (**c**)).

The real-time correlation function $G^{<}\left(t,s\right)$ can be evaluated from Equation (14) only with the aid of the noise models because of the required high circuit depth, implying an execution time longer than the dephasing time of superconducting circuits. To do so, we use the circuit in Equation (15) by collecting measurements for any pair of Pauli string operators $P_{\alpha }P_{\beta }$ (α,β=1,⋯,7) in Equation (14) and by setting the phase $\phi =0\;(\frac{\pi }{2})$ to select the imaginary (real) part of lesser contribution. In Figure 3, we collect our results for bare noisy simulations (dashed lines) to be compared to the exact noiseless simulation (solid lines). To interpret the results, we notice that, since particle densities ${\tilde{\nu }}^{\left(+,+\right)}\left(t\right)$ and ${\tilde{\nu }}^{\left(+,+\right)}\left(s\right)$ are Hermitian observables, they contribute only to the imaginary part of lesser terms, while the 49 terms in Equation (14) related to Pauli strings contribute to both the real and imaginary parts. This might explain the offset between the exact results and bare noise simulations we observe in the imaginary part and not in the real one. Indeed, we can conclude that this offset is caused by the higher deviation between exact and noisy measurements of the density $\langle {\tilde{\nu }}^{\left(+,+\right)}\left(t\right)\rangle$ as observed in Figure 2. On the other hand, the 49 Pauli strings pairs, that might contribute with opposite signs, yield a lower deviation. This offset is almost identical for the imaginary part in panels (a,c) for any coupling regime because of the same readout errors and a negligible value of particle density for low values of *s*. For higher values of *s*, the deviation decreases in stronger couplings, even if oscillations observed in the noiseless case are damped in the presence of noise.

**Figure 4:**
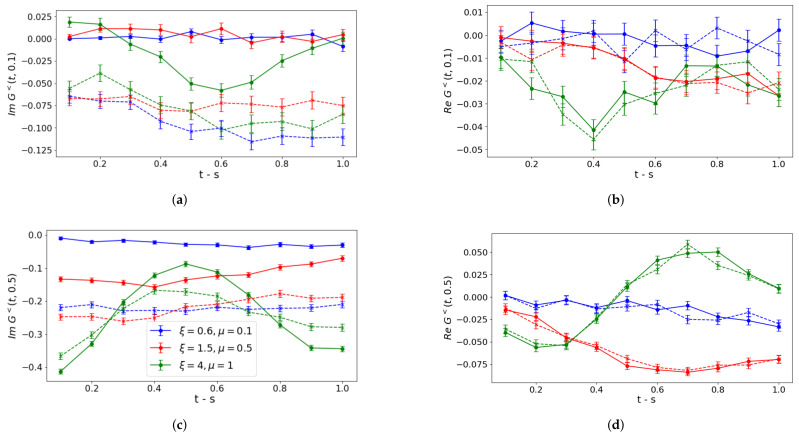
Simulations processed via T-REx mitigation. Lesser contribution to correlation functions of particle-densities ${\tilde{\nu }}^{\left(+,+\right)}\left(t\right)$ and ${\tilde{\nu }}^{\left(+,+\right)}\left(s\right)$ for a non-vanishing time *s*: s=0.1 in (**a**,**b**), s=0.5 in (**c**,**d**). Circuits are executed in qasm_simulator. Solid lines correspond to noiseless Trotter evolution with error bars caused by the finite number of shots, while dashed lines include noise models. Different colors refer to different coupling regimes (see panel (**c**)).

**Figure 5:**
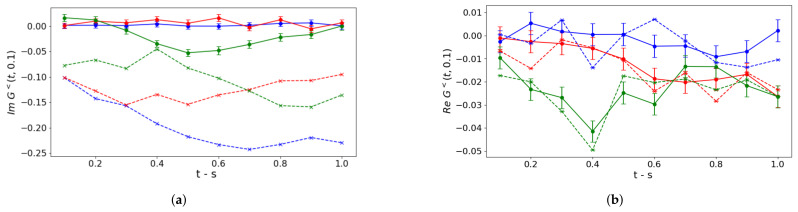

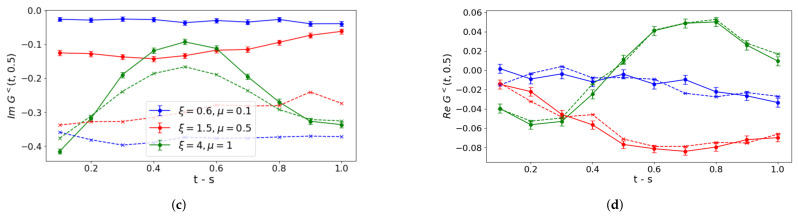
Simulations processed via ZNE mitigation. Lesser contribution to correlation functions of particle-densities ${\tilde{\nu }}^{\left(+,+\right)}\left(t\right)$ and ${\tilde{\nu }}^{\left(+,+\right)}\left(s\right)$ for a non-vanishing time *s*: s=0.1 in (**a**,**b**), s=0.5 in (**c**,**d**). Circuits are executed in qasm_simulator. Solid lines correspond to noiseless Trotter evolution with error bars caused by the finite number of shots, while dashed lines include noise models. Different colors refer to different coupling regimes (see panel (**c**)).

In Figure 4 and Figure 5, we collect our results for bare noisy simulation data corrected via T-REx and ZNE mitigation, respectively.

As for the former, we notice that real parts of lesser contributions shown in panels (b,d) are not distinguishable from the noiseless case, thus revealing a significant improvement with respect to the unmitigated case. Instead, the imaginary parts in panels (a,c) are almost unchanged because the dominant contribution comes from ${\tilde{\nu }}^{\left(+,+\right)}\left(t\right)$ and ${\tilde{\nu }}^{\left(+,+\right)}\left(s\right)$, which coincides in the noisy and mitigated scenario. The weak coupling regime oscillation is well captured with respect to its period in the considered time window, but the convergence towards the maximally mixed state causes amplitude dampings already observed in Figure 3.

Similar conclusions can be drawn concerning the real part of lesser contributions corrected by means of ZNE mitigation techniques, as it is shown in panels (b,d) of Figure 5, which show significantly improved behavior. Instead, a non-negligible worsening emerges in the imaginary parts as shown in panels (a,c), with the exception of the weak coupling oscillation in panel (c), which is almost unchanged. These behaviors reveal that the circuit folding procedure used in ZNE and discussed in Appendix F yields a non-trivial noise scaling, beyond the linear one, for the considered evaluation of the correlation function imaginary part. For sufficiently low values assumed by $G^{<}\left(t,s\right)$, errors behave as a small perturbation and are efficiently taken under control by mitigation procedures adopting the previously mentioned available LinearExtrapolator. Such effects are instead washed out for higher values of the considered function, with a correspondingly increasing order of magnitude for errors.

In general, the measured offset in imaginary parts is caused by an increasing number of contributions driven by mixed states, as explained by Equation (A26) in Appendix E, which bring additional contributions with respect to the targeted pure initial Dirac vacuum. A more precise description of output states requires an extension in terms of channels corresponding to gates, in order to target the generation of correlated noise [35], but this goes beyond the scope of this paper.

## 6. Conclusions and Outlook

We considered real-time dynamics of the Schwinger model on a periodic lattice in (1+1) dimensions, implemented on IBM Quantum [55] real devices and examined via different noise models. We proposed a quantum–classical approach to minimize the required computational cost: first we used translation by two lattice sites and charge conjugation symmetries to restrict the dynamics to the sector of the Dirac vacuum, then we reduced the number of triple-qubit gates involved in the Trotter evolution by choosing the optimal permutation of states in the qubits embedding. We examined first the real-time evolution of the particle-density operator in different coupling regimes, showing a resilient behavior with respect to noise in the weak coupling case. In Appendix B, we list the contribution to three qubits gates from the hopping term: in the strong coupling regime, these high-depth Trotter circuits act almost uniquely as a noise source without an effective contribution to the time evolution because of the low value of ξ. Then, we calculated the time-dependent correlation functions, which are very much affected by noise. Our results prove that error mitigation works properly for small variations in the measured real parts of lesser Green functions.

Further developments will investigate to what extent decoherence and noise resiliency might affect the analysis of errors occurrence in different coupling regimes [56], by explicitly identifying circuits parts more affected by a specific parameter. Non linear extrapolation is included in the new version of the package qiskit-ibm-runtime, and we will target the exploitation of this error mitigation resource.

## Figures and Tables

**Figure A1:**
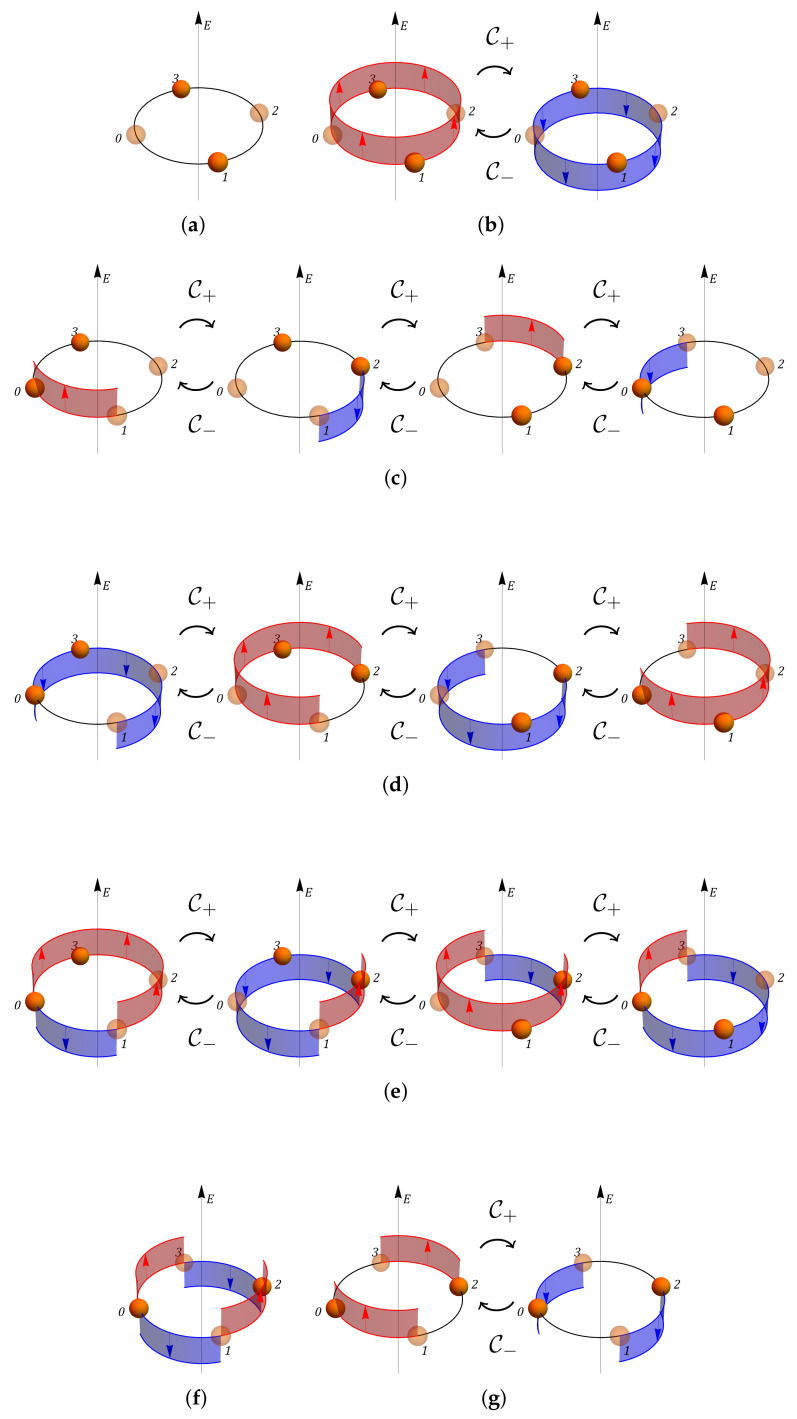
A basis of gauge-invariant states for the periodic lattice with N=4. (**a**,**b**) Dirac vacuum states; (**c**–**e**) one-meson states; (**f**,**g**) two-meson states. The action of the charge conjugation operators $C_{\pm }$ and the corresponding orbits are shown. In all panels, full (transparent) spheres represent occupied (empty) matter sites, while red (blue) edges correspond to a positive (negative) electric field on a link.

**Figure A2:**
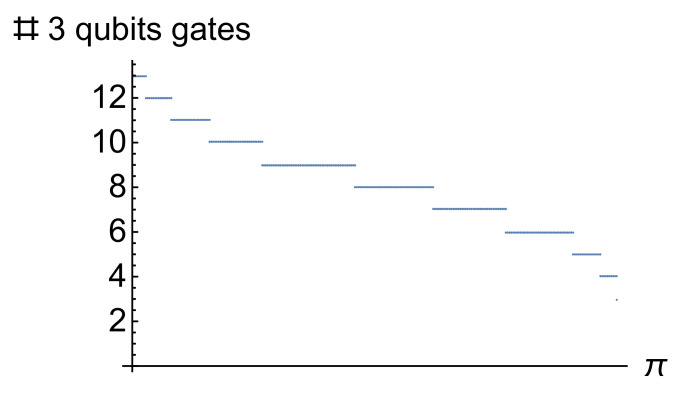
Number of triple-qubits gates implied by the embedded Hamiltonian decomposition (9) with respect to any permutation $\pi \in S_{8}$. Permutations are ordered with respect to the decreasing number of gates.

**Figure A3:**
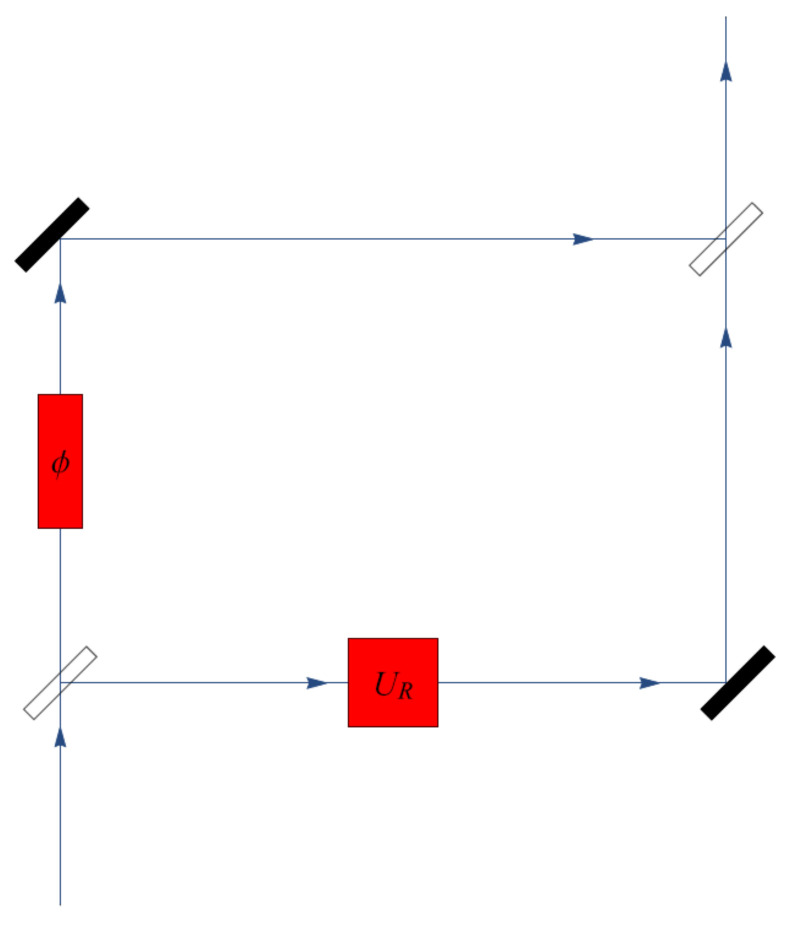
A Mach–Zender interferometer with mirrors and beam splitters represented by black and white rectangles, respectively. The implementation of a control unitary is referred to in red blocks.

## Data Availability

The raw data supporting the conclusions of this article will be made available by the authors on request.

## Abbreviations

The following abbreviations are used in this manuscript:

NISQ	noise intermediate-scale quantum
QED	quantum electrodynamics
DQPT	dynamical quantum phase transition
T-REx	twirled readout error extinction
ZNE	zero noise extrapolation

## Appendix A. Basis of the Gauge-Invariant Subspace

In the following, we consider a lattice of N=4 links, with two particles, and discretize the unitary group with $Z_{3}$. A state is labeled with the values $e_{k}=+,0,-$ of the electric field on the four links: $|e_{0}\;e_{1}\;e_{2}\;e_{3}\rangle$. Out of the possible $3^{4}$ configurations, only 18 are gauge-invariant, yielding a basis for the physical subspace. The latter can be split into disjoint orbits of the charge conjugation and translation operators. We notice that in this particular case [24], $T_{2}=C_{+}^{2}=C_{-}^{2}$, so in order to classify orbits, we can simply look at the action of the charge conjugation operator $C_{+}$.

All 18 states are shown in Figure A1, where we have the following:

1. Panels (a,b) show the Dirac vacuum, corresponding to the two even sites being empty and the two odd sites being filled, with the three possible total background fields, which are completely fixed by the value of the electric field $e_{0}$ on the edge joining the sites 0 and 1: the state with zero electric field background is a fixed point with respect to $C_{+}$, while the other two states with non-zero electric field background give one orbit.
2. Panels (c,d,e) show the 12 one-meson states, corresponding to occupying one even and one odd site, the three panels corresponding to the orbits of the three possible configurations obtained from the three possible Dirac vacuum configurations, respectively, by moving a particle from site 2 to site 3;
3. Panels (f,g) show the two-meson states, corresponding to the two even sites being filled and the two odd sites being empty and the three possible total field configurations, the two states with $|e_{0}\rangle =|0\rangle ,|+\rangle$ represent an orbit, while the state with $|e_{0}\rangle =|-\rangle$ is a fixed point.

In order to achieve an optimal encoding, we exploit the translation and charge conjugation symmetry operators that commute with the Hamiltonian, $T_{2}HT_{2}=H$ and $C_{\pm }HC_{\mp }=H$, to define the subspace to which the dynamics is restricted. The elements of a basis for such subspaces are obtained as linear combinations of the states shown in Figure A1 as follows.

- subspace with $T_{2}=+1;\;C_{+}=+1$:
  (A1) $$\begin{array}{c} |\psi_{1}\rangle =|0000\rangle , \end{array}$$
  (A2) $$\begin{array}{l} |\psi_{2}\rangle =\frac{1}{2}\left(|+000\rangle +|000-\rangle +|0-00\rangle +|00+0\rangle \right), \end{array}$$
  (A3) $$\begin{array}{cc} \; & |\psi_{3}\rangle =\frac{1}{\sqrt{2}}\left(|+0+0\rangle +|0-0-\rangle \right), \end{array}$$
  (A4) $$\begin{array}{cc} \; & |\psi_{4}\rangle =\frac{1}{2}\left(|+0++\rangle +|+++0\rangle +|--0-\rangle +|0---\rangle \right), \end{array}$$
  (A5) $$\begin{array}{cc} \; & |\psi_{5}\rangle =\frac{1}{\sqrt{2}}\left(|++++\rangle +|----\rangle \right), \end{array}$$
  (A6) $$\begin{array}{cc} \; & |\psi_{6}\rangle =\frac{1}{2}\left(|-+++\rangle +|++-+\rangle +|---+\rangle +|-+--\rangle \right), \end{array}$$
  (A7) $$\begin{array}{cc} \; & |\psi_{7}\rangle =|-+-+\rangle ; \end{array}$$
- subspace with $T_{2}=+1;\;C_{+}=-1$:
  (A8) $$\begin{array}{cc} \; & |\psi_{8}\rangle =\frac{1}{2}\left(|+000\rangle -|000-\rangle -|0-00\rangle +|00+0\rangle \right), \end{array}$$
  (A9) $$\begin{array}{cc} \; & |\psi_{9}\rangle =\frac{1}{\sqrt{2}}\left(|+0+0\rangle -|0-0-\rangle \right), \end{array}$$
  (A10) $$\begin{array}{cc} \; & |\psi_{10}\rangle =\frac{1}{2}\left(|+0++\rangle +|+++0\rangle -|--0-\rangle -|0---\rangle \right), \end{array}$$
  (A11) $$\begin{array}{cc} \; & |\psi_{11}\rangle =\frac{1}{\sqrt{2}}\left(|++++\rangle -|----\rangle \right), \end{array}$$
  (A12) $$\begin{array}{cc} \; & |\psi_{12}\rangle =\frac{1}{2}\left(|-+++\rangle +|++-+\rangle -|---+\rangle -|-+--\rangle \right); \end{array}$$
- subspace with $T_{2}=-1;\;C_{+}=+i$:
  (A13) $$\begin{array}{cc} \; & |\psi_{13}\rangle =\frac{1}{2}\left(|+000\rangle +i|000-\rangle -i|0-00\rangle -|00+0\rangle \right), \end{array}$$
  (A14) $$\begin{array}{cc} \; & |\psi_{14}\rangle =\frac{1}{2}\left(|+0++\rangle -|+++0\rangle -i|--0-\rangle +i|0---\rangle \right), \end{array}$$
  (A15) $$\begin{array}{cc} \; & |\psi_{15}\rangle =\frac{1}{2}\left(|-+++\rangle -|++-+\rangle +i|---+\rangle -i|-+--\rangle \right); \end{array}$$
- subspace with $T_{2}=-1;\;C_{+}=-i$:
  (A16) $$\begin{array}{cc} \; & |\psi_{16}\rangle =\frac{1}{2}\left(|+000\rangle -i|000-\rangle +i|0-00\rangle -|00+0\rangle \right), \end{array}$$
  (A17) $$\begin{array}{cc} \; & |\psi_{17}\rangle =\frac{1}{2}\left(|+0++\rangle -|+++0\rangle +i|--0-\rangle -i|0---\rangle \right), \end{array}$$
  (A18) $$\begin{array}{cc} \; & |\psi_{18}\rangle =\frac{1}{2}\left(|-+++\rangle -|++-+\rangle -i|---+\rangle +i|-+--\rangle \right). \end{array}$$

The Hamiltonian diagonal block presented in Equation (8) describes the dynamics in the sector spanned by eigenvectors in the subspace with $T_{2}=+1;\;C_{+}=+1$. The remaining ones in the diagonal blocks structure $UHU^{†}=H^{\left(+,+\right)}⊕H^{\left(+,-\right)}⊕H^{\left(-,+i\right)}⊕H^{\left(-,-i\right)}$ are not involved in the time evolution of the initial state that we fix to be the Dirac vacuum, but, for completeness, we show all of them:

(A19) $$H^{\left(+,-\right)}=\left(\begin{array}{ccccc} \frac{\pi }{3} & \frac{\xi }{\sqrt{2}} & \; & & \\ \frac{\xi }{\sqrt{2}} & 2\mu +\frac{2\pi }{3} & \frac{\xi }{\sqrt{2}} & \; & \\ \; & \frac{\xi }{\sqrt{2}} & \pi & \frac{\xi }{\sqrt{2}} & \\ \; & \; & \frac{\xi }{\sqrt{2}} & -2\mu +\frac{4\pi }{3} & \frac{\xi }{\sqrt{2}}\\ \; & \; & & \frac{\xi }{\sqrt{2}} & \frac{4\pi }{3} \end{array}\right),\;\;H^{\left(-,+i\right)}=H^{\left(-,-i\right)}=\mathrm{diag}\left\{\frac{\pi }{3},\pi ,\frac{4\pi }{3}\right\}.$$

## Appendix B. Optimal Permutation

With the simplest embedding, the Hamiltonian in the (+,+) sector is written according to Formula (9), where the following are the 20 non-zero coefficients:

$c_{\left(0,0,0\right)}=\frac{3\pi }{4}$	$c_{\left(0,0,1\right)}=\frac{(1+\sqrt{2})\xi }{4}$	$c_{\left(0,0,3\right)}=\frac{\pi }{12}$	$c_{\left(0,1,1\right)}=\frac{(2+\sqrt{2})\xi }{8}$	$c_{\left(1,1,1\right)}=\frac{\xi }{4\sqrt{2}}$
$c_{\left(0,3,0\right)}=-\mu$	$c_{\left(0,3,3\right)}=-\frac{\pi }{6}-\mu$	$c_{\left(0,3,1\right)}=\frac{\xi }{4}$	$c_{\left(0,2,2\right)}=\frac{(2+\sqrt{2})\xi }{8}$	$c_{\left(1,2,2\right)}=-\frac{\xi }{4\sqrt{2}}$
$c_{\left(3,0,0\right)}=-\frac{\pi }{4}$	$c_{\left(3,0,3\right)}=-\frac{\pi }{4}$	$c_{\left(3,0,1\right)}=\frac{\xi }{4}$	$c_{\left(3,1,1\right)}=\frac{(\sqrt{2}-2)\xi }{8}$	$c_{\left(2,1,2\right)}=\frac{\xi }{4\sqrt{2}}$
$c_{\left(3,3,0\right)}=-\frac{\pi }{3}$	$c_{\left(3,3,1\right)}=\frac{(\sqrt{2}-1)\xi }{4}$	$c_{\left(3,3,3\right)}=\frac{\pi }{6}$	$c_{\left(3,2,2\right)}=\frac{(\sqrt{2}-2)\xi }{8}$	$c_{\left(2,2,1\right)}=\frac{\xi }{4\sqrt{2}}$

The term with $c_{\left(0,0,0\right)}$ is just a phase, and it can be safely neglected. Then, there are 4 single-qubit, 8 two-qubit, and 7 three-qubit terms.

The form of the Hamiltonian can be considerably simplified if we modify the embedding by considering the optimal permutation of the indices $\pi_{o}=\left(7,6,1,2,4,5,8,3\right)$, which is characterized by the same phase term and the following non-zero coefficients:

$c_{\left(0,0,3\right)}=-\frac{\pi }{6}$	$c_{\left(1,0,0\right)}=\frac{\xi }{2\sqrt{2}}$	$c_{\left(1,0,3\right)}=-\frac{\xi }{2\sqrt{2}}$	$c_{\left(0,3,3\right)}=\frac{\pi }{12}+\mu$	$c_{\left(0,1,1\right)}=\frac{\xi }{4\sqrt{2}}$
$c_{\left(0,3,0\right)}=\frac{\pi }{2}$	$c_{\left(3,3,0\right)}=\frac{\pi }{12}+\mu$	$c_{\left(0,2,2\right)}=-\frac{\xi }{4\sqrt{2}}$	$c_{\left(0,0,1\right)}=\frac{(4+\sqrt{2})\xi }{8}$	$c_{\left(0,3,1\right)}=\frac{\xi }{4\sqrt{2}}$
$c_{\left(3,0,3\right)}=\frac{\pi }{12}$	$c_{\left(3,3,1\right)}=-\frac{\xi }{4\sqrt{2}}$	$c_{\left(3,1,1\right)}=-\frac{\xi }{4\sqrt{2}}$	$c_{\left(3,0,1\right)}=\frac{(4-\sqrt{2})\xi }{8}$	$c_{\left(3,2,2\right)}=\frac{\xi }{4\sqrt{2}}$

yielding 4 single-qubit, 8 two-qubit, and 3 three-qubit gates. For the problem under consideration, such optimal permutation can be simply obtained by testing all possible 8! permutations as shown in Figure A2, which gives the number of terms with a product of three-qubit gates, with permutations ordered from the maximum number to the lowest, which is 3 in this case.

With this permutation, the particle-density operator is given by Equation (11) with 8 non-zero coefficients given by $d_{\left(0,0,0\right)}=\frac{7}{16},d_{\left(0,3,3\right)}=\frac{5}{16},d_{\left(3,3,0\right)}=\frac{3}{16},d_{\left(0,3,0\right)}=d_{\left(3,0,0\right)}=d_{\left(3,0,3\right)}=\frac{1}{16},d_{\left(0,0,3\right)}=d_{\left(3,3,3\right)}=-\frac{1}{16}$. This allows to write

(A20) $${\tilde{\nu }}^{\left(+,+\right)}=d_{\left(0,0,0\right)}I+\sum_{\alpha =1}^{7}P_{\alpha },$$

where the seven operators $P_{\alpha }$ are given by the seven non-zero coefficients $d_{\left(i,j,k\right)}$ multiplied by the corresponding triple-string of Pauli operators $\sigma_{i}⊗\sigma_{j}⊗\sigma_{k}$ not equal to the identity.

## Appendix C. General Procedure for Classical–Quantum Embedding

For future research, it is important to design an algorithm to find the gauge-invariant subspace and obtain the optimal permutation that is able to perform fast also when we scale up the lattice size *N*. The basic procedure is as follows:

- Step 1: Store a list of possible site configurations for all even and odd sites. This is a total of six configurations each for both even and odd sites due to the following:
  - Each site, represented as *x*, can be filled or empty;
  - For either value of occupancy, the link to the right, represented as $e_{x,x+1}$, can take three possible values of electric field. The link to the left, $e_{x-1,x}$, is fixed by Gauss’ law.
- Step 2: Place sites 0,…N−1 one after the other, following the pattern even–odd–even–odd… ensuring that the values of the common links match.
- Step 3: Impose periodic boundary conditions: $e_{N,N+1}=e_{-1,0}$.
- Step 4: Eliminate the configurations that do not correspond to the right total number of particles (in our case, half filling). After this step, we obtain the basis states which are best represented as a dictionary, with each site mapping to its occupancy (0 or 1), and each link mapping to a $Z_{3}$ element representing the electric field on that link (with values −1,0,+1).
- Step 5: Arrange states into multiplets under translation and charge conjugation symmetries and isolate the multiplet containing the vacuum state. This multiplet corresponds to the (+,+) sector and evolves independently of the other blocks.
- Step 6: Find the Hamiltonian $H\equiv H^{\left(+,+\right)}$ matrix elements in this multiplet. Embed this matrix into a $2^{M}$-dimensional space, where *M* is the lowest such value the matrix can be embedded into. This gives the number of needed qubits.
- Step 7: Find an optimal permutation of the $2^{M}$ basis states such that the number of higher-qubit gates in the Pauli gate expansion of H is minimized.
- Step 8: Use the Pauli gate expansion of the Hamiltonian in the optimized encoding to construct the Trotter steps in the time evolution.

For large system sizes, it is unfeasible to perform Step 7 using a brute-force approach like we performed for the case of N=4. This is a discrete optimization problem where we have to find the optimal permutation of basis states. The entire search space is M!, which grows faster than exponential, and it quickly becomes unfeasible to perform an exhaustive search.

The approach we take is a local search algorithm, which can be outlined as follows:

- We define the objective function as
  (A21) $$F\left(H\right)=\sum_{i}Q\left[\mathrm{Tr}\left(HX_{i}\right)\right],\;Q\left(x\right)\equiv \left\{\begin{array}{cc} 1, & \mathrm{if}\;x\neq 0\\ 0, & \mathrm{otherwise} \end{array}\right.,$$
  where $\left\{X_{i}\right\}$ is the set of *M*-qubit Pauli gates. This simply counts the number of such gates in the decomposition of the Hamiltonian H.
- Start with an initial configuration of the Hamiltonian $H_{init}$ and store $f_{opt}=F\left(H_{init}\right)$.
- We choose a “neighborhood” of a configuration of H to be the set of configurations related to it by a single swap of two indices: row i⟷ row *j*, column i⟷ column *j*. For a $d=2^{M}$-dimensional system, the dimension of each neighborhood scales as $O(d^{2})$.
- Go to a locally optimal configuration in this neighborhood, according to some heuristic choice; calculate the objective function for the new configuration and update $f_{best}$.
- Continue until $f_{best}$ cannot be improved any further.

We tested this procedure with a few different lattice sizes N=2,4,6,8. For these cases, we simply used a greedy local search algorithm [57]. The optimal number of *M*-qubit gates found and the runtimes are listed in Table A1 below.

**Table A1:** Results of typical runs of the greedy algorithm.

*L*	System Size *M*	*M*-Qubit Gates in Optimal Permutation	Runtime
2	4	2	$∼10^{-3}$ s
4	8	3	$∼10^{-2}$ s
6	16	8	$∼10^{-1}$ s
8	64	123	$∼10^{3}$ s

This local search algorithm gives us a “good enough” solution, but there is no way of knowing if it is the true optimal solution. We can improve our results by running the algorithm a few times from random initial configurations and taking the best result.

## Appendix D. Digital Circuit for Trotter Evolution

We describe here the Trotter expansion of the evolution operator U(t) corresponding to a time step Δt of the optimal permuted Hamiltonian H, framed in qiskit notation. The decomposition in simpler operators proceeds as follows.

- Single-qubit gates are related to the coefficients with $c_{\left(0,0,1\right)},c_{\left(0,0,3\right)},c_{\left(0,3,0\right)},c_{\left(1,0,0\right)}$. They can implemented by the circuit
  which is endowed with a high average fidelity.
- Two-qubit gates can be grouped in three different operators. The first one contains the terms corresponding to the coefficients $c_{\left(0,1,1\right)},c_{\left(0,2,2\right)},c_{\left(0,3,3\right)}$ and is represented by the circuit
  The second operator contains the terms corresponding to the coefficients $c_{\left(0,3,1\right)},c_{\left(1,0,3\right)}$ and is given by the circuit
  Finally the third operator, which contains the terms with the coefficient $c_{\left(3,0,1\right)},c_{\left(3,0,3\right)}$, $c_{\left(3,3,0\right)}$ corresponds to the circuit
  Here, the abundance of controlled gates causes a decreasing average fidelity with respect to the single-qubits gates case.
- Three-qubit gates are grouped in two different operators, where the first one contains the terms with coefficients $c_{\left(3,3,1\right)}$ and is given by the circuit
  while the second contains the terms with coefficients $c_{\left(3,1,1\right)},c_{\left(3,2,2\right)}$ and is implemented by the circuit

The composition of all these pieces yields the total circuit that represents a single Trotter step evolution. After further simplification emerging from the gate composition and available commutations, we obtain the circuit of Figure 1.

## Appendix E. The Circuit of Mach–Zender Interferometer

The circuit presented in Section 4 to evaluate correlation functions is based on the Mach–Zender interferometer [53,54]. The setup is referred to in Figure A3, where arrows identify input and output ports. White and black rectangles represent balanced beam splitters and mirrors, respectively. These devices act on the ancilla *A* degree of freedom $H_{A}=\mathrm{span}\left\{|0\rangle ,|1\rangle \right\}$ related with wave packets moving along vertical and horizontal directions. We include internal degrees of freedom associated with our register *R* according to the tensor product $H_{A}⊗H_{R}$ such that the input state is $\varrho_{\mathrm{in}}=|0\rangle \langle 0|⊗\varrho_{R}$, elaborated by mirrors $U_{M}=X⊗1$, beam splitters $U_{B}=H⊗1$ and a controlled gate

(A22) $$U=\left(\begin{array}{cc} e^{i\phi } & 0\\ 0 & 0 \end{array}\right)⊗1+\left(\begin{array}{cc} 0 & 0\\ 0 & 1 \end{array}\right)⊗U_{R}.$$

The output state is defined by the transformation

(A23) $$\varrho_{\mathrm{in}}\to \varrho_{\mathrm{out}}=U_{B}U_{M}UU_{B}\varrho_{\mathrm{in}}U_{B}^{†}U^{†}U_{M}^{†}U_{B}^{†},$$

that yields the following expression:

(A24) $$\begin{array}{cc} \varrho_{\mathrm{out}}=\frac{1}{4} & \left[\left(\begin{array}{cc} 1 & 1\\ 1 & 1 \end{array}\right)⊗U_{R}\varrho_{R}U_{R}^{†}+\left(\begin{array}{cc} 1 & -1\\ -1 & 1 \end{array}\right)⊗\varrho_{R}\right.\\ \; & \left.\;\;+e^{i\phi }\left(\begin{array}{cc} 1 & 1\\ -1 & -1 \end{array}\right)⊗\varrho_{R}U_{R}^{†}+e^{-i\phi }\left(\begin{array}{cc} 1 & -1\\ 1 & -1 \end{array}\right)⊗U_{R}\varrho_{R}\right]. \end{array}$$

The resulting intensity along the vertical direction is obtained by projecting the output state

(A25) $${\mathrm{Tr}}_{AR}\left\{\left(|0\rangle \langle 0|⊗1\right)\varrho_{\mathrm{out}}\right\}=\frac{1}{2}\left(1+|{\mathrm{Tr}}_{R}\left\{U_{R}\varrho_{R}\right\}|\cos\left(\phi -\arg\left({\mathrm{Tr}}_{R}\left\{U_{R}\varrho_{R}\right\}\right)\right)\right),$$

where we use ${\mathrm{Tr}}_{R}\left\{\varrho_{R}U_{R}^{†}\right\}={\mathrm{Tr}}_{R}{\left\{U_{R}\varrho_{R}\right\}}^{*}$ in order to introduce the visibility [53] of the interference pattern $v=|{\mathrm{Tr}}_{R}\left\{U_{R}\varrho_{R}\right\}|$. For a register in a pure state $|\psi_{0}\rangle$, the visibility reads $v=|\langle \psi_{0}\left|U_{R}\right|\psi_{0}\rangle |$, while in a general mixed state $\varrho_{R}=\sum_{k}w_{k}|k\rangle \langle k|$,

(A26) $$v=\left|\sum_{k}w_{k}v_{k}e^{i\phi_{k}}\right|,$$

with $v_{k}=\left|\langle k|U_{R}|k\rangle \right|$ and $\phi_{k}=\arg\left(\langle k|U_{R}|k\rangle \right)$ [53].

The diagonal elements of the Mach–Zender interferometer output in Figure A3 are analogous to the ones of the output of the circuit [58]

yielding an opposite sign for off-diagonal elements with respect to Equation (A24):

(A27) $$\begin{array}{cc} \varrho_{\mathrm{out}}=\frac{1}{4} & \left[\left(\begin{array}{cc} 1 & -1\\ -1 & 1 \end{array}\right)⊗U_{R}\varrho_{R}U_{R}^{†}+\left(\begin{array}{cc} 1 & 1\\ 1 & 1 \end{array}\right)⊗\varrho_{R}\right.\\ \; & \left.\;\;+e^{i\phi }\left(\begin{array}{cc} 1 & -1\\ 1 & -1 \end{array}\right)⊗\varrho_{R}U_{R}^{†}+e^{-i\phi }\left(\begin{array}{cc} 1 & 1\\ -1 & -1 \end{array}\right)⊗U_{R}\varrho_{R}\right]. \end{array}$$

followed by the measurement

(A28) $${\mathrm{Tr}}_{AR}\left\{\left(Z⊗1\right)\varrho_{\mathrm{out}}\right\}=\left|{\mathrm{Tr}}_{R}\left\{U_{R}\varrho_{R}\right\}\right|\cos\left(\phi -\arg({\mathrm{Tr}}_{R}\left\{U_{R}\varrho_{R}\right\})\right).$$

## Appendix F. T-REx and ZNE Mitigation Schemes

In this Appendix section, we give a brief description of the two error mitigation techniques used in the paper.

The first method consists in T-REx, which focuses on readout errors [48]. An operator *V* acts on a system of *N* qubits, modifying its state as follows:

(A29) $$\varrho_{\mathrm{in}}\to \varrho_{\mathrm{out}}=V\varrho_{\mathrm{in}}V^{†},$$

that we have to sample with respect to possible output strings $x\in Z_{2}^{N}$ by means of a positive operator-valued measure (POVM) $E_{x}=|x\rangle \langle x|$. We assume that a noise map *A* affects measurements, representing a $2^{N}\times 2^{N}$ left-stochastic matrix, which yields a noisy readout distribution $\tilde{p}=Ap$: the element $A_{x,y}=\langle x|A|y\rangle$ quantifies the probability of measuring *y* instead of *x*, defining a noisy POVM ${\tilde{E}}_{x}=\sum_{y}A_{x,y}|y\rangle \langle y|$.

Given a string $s\in Z_{2}^{N}$, it is possible to define

(A30) $$Z_{s}=\sum_{x}{\left(-1\right)}^{\langle s,x\rangle }|x\rangle \langle x|,\;X_{s}=\sum_{x}|x+s\rangle \langle x|=\sum_{x}|x\rangle \langle x+s|=X_{s}^{†},$$

involved in the targeted estimation of

(A31) $$\langle Z_{s}\rangle =\mathrm{Tr}\left\{Z_{s}\varrho_{\mathrm{out}}\right\}=\sum_{x}{\left(-1\right)}^{\langle s,x\rangle }\mathrm{Tr}\left\{E_{x}\varrho_{\mathrm{out}}\right\}$$

whose unmitigated noisy version is $\langle {\tilde{Z}}_{s}\rangle =\sum_{x}{\left(-1\right)}^{\langle s,x\rangle }\mathrm{Tr}\left\{{\tilde{E}}_{x}\varrho_{\mathrm{out}}\right\}$. The mitigation of this readout error is achieved by applying random bit flips before and after the noisy measurements as expressed by the twirled noise map and associated POVM

(A32) $$A^{*}=\frac{1}{2^{N}}\sum_{s}X_{s}AX_{s}^{†},\;{\tilde{E}}_{x}^{*}=\sum_{y}A_{x,y}^{*}|y\rangle \langle y|.$$

The eigensystem of this map is obtained by exploiting the definition of the eigenvectors $|v_{w}\rangle =\sum_{x}{\left(-1\right)}^{\langle w,x\rangle }|x\rangle$ such that the eigenvalue equation is deduced

(A33) $$\begin{array}{cc} A^{*}|v_{w}\rangle = & \;\frac{1}{2^{N}}\sum_{s,x}\sum_{a,b}{\left(-1\right)}^{\langle w,x\rangle }A_{a,b}|a+s\rangle \langle b+s|x\rangle \\ = & \;\frac{1}{2^{N}}\sum_{x}\sum_{a,b}{\left(-1\right)}^{\langle w,x+a+b\rangle }A_{a,b}|x\rangle =\lambda_{w}|v_{w}\rangle , \end{array}$$

yielding $\lambda_{w}=\frac{1}{2^{N}}\sum_{a,b}{\left(-1\right)}^{\langle w,a+b\rangle }A_{a,b}$. The twirled noise expectation reads

(A34) $$\begin{array}{cc} \langle {\tilde{Z}}_{w}^{*}\rangle = & \;\sum_{x}{\left(-1\right)}^{\langle w,x\rangle }\mathrm{Tr}\left\{{\tilde{E}}_{x}^{*}\varrho_{\mathrm{out}}\right\}=\sum_{x,y}{\left(-1\right)}^{\langle w,x\rangle }\langle x|A^{*}|y\rangle \mathrm{Tr}\left\{|y\rangle \langle y|\varrho_{\mathrm{out}}\right\}\\ = & \;\sum_{y}\langle v_{w}\left|A^{*}\right|y\rangle \mathrm{Tr}\left\{|y\rangle \langle y|\varrho_{\mathrm{out}}\right\}=\lambda_{w}\langle Z_{w}\rangle , \end{array}$$

which for the state $\varrho_{\mathrm{out}}=|0\rangle \langle 0|$ allows us to estimate $\langle {\tilde{Z}}_{w}^{*}\rangle =\lambda_{w}$ since $\langle Z_{w}\rangle =1$. The same quantity is used to obtain noise mitigation for other values of $\varrho_{\mathrm{out}}$.

The second technique refers to ZNE, adopting a noise scaling in order to extrapolate noiseless expectation values [49]. In practice, a “de-optimizing” procedure is applied by increasing the circuit depth. We assume that the action of a circuit, as expressed in Equation (A29), is composed of *d* unitary layers

(A35) $$V=L_{d}L_{d-1}\ldots L_{2}L_{1},$$

such that the depth is equal to *d*. A first step aimed at noise scaling consists in unitary folding, which replaces the unitary circuit by

(A36) $$V\to V{\left(V^{†}V\right)}^{n},$$

where *n* is a positive integer leading to a new depth (2n+1)d, with no logical effect since $V^{†}V=1$. In order to improve the scaling resolution, it is possible to exploit also a partial folding concerning the last *s* layers of the circuit

(A37) $$V\to V{\left(V^{†}V\right)}^{n}L_{d}^{†}L_{d-1}^{†}\ldots L_{d-s}^{†}L_{d-s}\ldots L_{d-1}L_{d},$$

whose depth is (2n+1)d+2s. The depth stretching d→λd is ruled by a scale resolution 2/d

(A38) $$\lambda =1+\frac{2k}{d},$$

with k=1,2,…,nd+s. We assume a linear, polynomial, or exponential dependence of the observables’ expectation values with respect to the scaling parameter 〈O(λ)〉, where 〈O(1)〉 is the natural noise, and we extrapolate the noiseless case 〈O(0)〉.
